# Effects of yeast hydrolysate supplementation on intestinal morphology, barrier, and anti-inflammatory functions of broilers

**DOI:** 10.5713/ab.21.0374

**Published:** 2022-01-04

**Authors:** Ting Wang, Kang Cheng, QiMing Li, Tian Wang

**Affiliations:** 1 College of Animal Science and Technology, Nanjing Agricultural University, Nanjing, Jiangsu 210095, China

**Keywords:** Anti-inflammation, Broiler, Intestinal Barrier, Intestinal Morphology, Yeast Hydrolysate

## Abstract

**Objective:**

This study was conducted to evaluate the effects of dietary yeast hydrolysate (YH) supplementation on intestinal morphology, barrier, and anti-inflammatory functions of broilers.

**Methods:**

A total of 320 one day old male broilers were randomly allocated into four groups with eight replicates of ten broilers each. The broilers were supplemented with a basal diet (the control group) or basal diets adding 50, 100, 150 mg/kg YH, respectively. This trial lasted for 42 days. The orthogonal polynomial contrasts were used to determine the linear and quadratic effects of increasing levels of YH.

**Results:**

In our previous research, supplementing YH improved growth performance by enhancing body weight gain but decreased feed-to-gain ratio. In this study, compared with the control group, dietary YH addition linearly and quadratically decreased serum diamine oxidase activity (p<0.05). Additionally, supplementing YH linearly and/or quadratically decreased jejunal crypt depth (CD), tumor necrosis factor-alpha (TNF-α) concentration as well as mucin 2, interleukin-6 (IL-6), IL-1β, TNF-α, nuclear factor kappa B, and myeloid differentiation factor 88 gene expression levels (p<0.05). Whereas the jejunal villus height (VH), VH/CD, IL-10 concentration as well as zonula occludens-1 and *IL-10* gene expression levels were linearly and/or quadratically increased by YH supplementation (p<0.05).

**Conclusion:**

Dietary YH supplementation improved intestinal morphology, barrier and anti-inflammatory functions while decreased intestinal permeability of broilers, which might be related with altering pertinent genes expression. This study provides evidence of YH as a promising feed additive for broilers.

## INTRODUCTION

Small intestine, as the barrier between the body and external environment, is easily affected or even damaged by various adverse factors including feed toxin, pathogenic bacteria, invasive viruses, high temperature and unsanitary feeding conditions. A well-developed intestinal mucosa and barrier integrity are beneficial to decrease intestinal permeability and defend against external disadvantages, thus maintaining the health of the intestine and improving growth performance of animals [1,2]. Dietary manipulation is an effectual way of shaping intestinal mucosal development and barrier function [2]. Thus, it is essential to improve intestinal barrier integrity and anti-inflammatory function though nutritional efforts, thus improving intestinal health status and growth performance, which is of great significance for poultry production.

Various yeast-derived products including yeast culture, yeast extracts, yeast hydrolysate (YH) and yeast cell wall, are natural feed additives which have been applied in animal production for many years [3–9]. Dietary supplementation with whole yeast and yeast cell wall showed positive effects on pancreatic enzyme activities and ileal protein digestibility, thus improving growth performance and meat yield of broiler chickens [10]. Bilal et al [11] indicated that yeast-based additives could improve humoral immunity by increasing serum immunoglobulin A level. Besides, yeast additives are probably supportive of immune hemostasis though medicating the balance between pro- and anti-inflammatory activities. Recent studies have found that yeast cell wall addition to feed improved serum immune response as well as intestinal integrity and immune function of animals [8,12]. Moreover, fractions or extracts of yeast cell wall including β-glucan and mannan-oligosaccharide (MOS) could improve intestinal barrier, anti-inflammatory and immune functions as well as regulate the microflora in the hindgut in different animals [13–15].

Yeast hydrolysate is derived from *Saccharomyces cerevisiae*, which has a lower cost advantage compared with other extracted yeast additives [7]. Fu et al [16] demonstrated that supplementing YHs enhanced growth performance, serum immune cytokines levels and beneficial bacteria in the cecum of growing-finishing pigs. Also, dietary brewer’s YH addition improved growth performance and the digestibility of nutrients in growing pigs [7]. In addition, supplementing YH increased antioxidant capacity as well as disease resistance and non-specific immunity in aquatic animals [17,18]. It follows that the application of YH in animal production has great value and potential. However, the existing literature on YH has mainly studied ruminants, pigs or aquatic animals, little information is available regarding the effects of YH supplementation in broilers, especially in terms of intestinal development and health. According to our previous study, we have found that YH supplementation had beneficial effects on growth performance, intestinal antioxidant, and immune functions of broilers [19,20]. To explore further, this study aimed to evaluate different levels of YH addition on intestinal morphology, barrier, and anti-inflammatory functions of broilers, which might provide evidence for the scientific application of YH in broilers production.

## MATERIALS AND METHODS

All experimental design and procedures were supervised by Institutional Animal Care and Use Committee of Nanjing Agricultural University following the requirements of the Regulations for the Administration of Affairs Concerning Experimental Animals of China (NJAU-CAST-2017-019).

### Experimental design and animal management

A total of 320 male one-day-old Arbor Acres broiler chicks (average body weight, 39.50±0.30 g) were purchased from a commercial hatchery (Yantai Land Animal Husbandry Co. Ltd., Shandong, China) and randomly assigned into four treatments for a 42-d feeding trial. Each treatment had eight replicates and each replicate had ten birds. Birds in the control group (CON) were provided with a basal diet, and the other three groups were fed the basal diet with the addition of 50, 100, and 150 mg/kg YH respectively. The YH was obtained from Church & Dwight Co., Inc. (Princeton, NJ, USA), which is a complex product derived from enzymatic hydrolysis of the yeast and mainly composed of crude protein (>30%), functional polysaccharides (MOS >6%; β-glucan >8%), yeast culture and nucleotides. All diets were formulated to satisfy or exceed the nutritional requirements according to the National Research Council (1994) [21]. The composition and nutrition levels of all diets are presented in Table 1. All broiler chickens were raised in three-level cages (120 ×60×50 cm) and given free access to feed and water. The environmental temperature in the house was controlled ranged from 34°C to 36°C during 1 to 7 d and subsequently declined to a final temperature of 24°C until the end of the experiment.

### Sampling

On 21 d and 42 d, eight birds (one bird per pen) from each group were randomly selected after a 12-h feed deprivation. Heparinized blood collected from the wing vein was centrifuged at 4,000×g for 15 min at 4°C to collect top serum samples, which were then stored at −80°C until analysis. After blood collection, birds were euthanized by cervical dislocation and necropsied. The jejunal tissues were rapidly collected and the jejunal sections about 2 cm in length were sliced at the middle position and fixed in chilled 4% paraformaldehyde for morphometric evaluation. The remaining jejunum segments were opened longitudinally at the same position and flushed the residual digesta with ice-cold phosphate buffer solution to collect mucosal samples. The jejunal mucosa was collected by directly scraping using a sterile glass microscope slide at 4°C, which were then immediately frozen in liquid nitrogen and stored at −80°C until analysis.

### Determination of serum diamine oxidase activity

The serum diamine oxidase (DAO) activity was determined by a commercial diagnostic kit purchased from Nanjing Angle Gene (Jiangsu Province, China). The experimental operations were strictly complied with the instructions of the manufacturer.

### Determination of jejunal morphology

The specimens of the jejunal tissues were first fixed in a paraformaldehyde solution for 24 h at room temperature. After that, they were dehydrated through an upgraded series of ethanol and xylene soaking, and then were embedded in paraffin blocks. Cross sections of the tissue segments were sliced at a thickness of 5 μm and stained with hematoxylin and eosin. The villus height (VH, from the tip of villus to the villus-crypt junction level for 10 villi per section) and crypt depth (CD, the vertical distance from the villus-crypt junction to the lower limit of the crypt for 10 corresponding crypts per section) were measured using a light microscope equipped with a computer-assisted morphometric system (Nikon Corporation, Tokyo, Japan). The ratio between VH and CD was also calculated (VH/CD).

### Determination of jejunal inflammatory cytokines

About 1 g jejunal mucosal samples were cut off and added with ice-cold sodium chloride solution (154 mmol/L), then homogenized (1:4, wt/vol) using an ultraturrax homogenizer (Tekmar Co., Cinatti, OH, USA). Afterwards, the above homogenate was centrifuged at 4,000×*g* for 15 min at 4°C. The top supernatant was promptly collected and used for assaying jejunal mucosal inflammatory cytokines.

The chicken-specific enzyme-linked immuno sorbent assay quantification kits (Angle Gene, Nanjing, China) were used to measure the concentrations of tumor necrosis factor α (TNF-α) and interleukin 10 (IL-10) in the jejunal mucosal samples based on the manufacturer’s instructions. The total protein level of each sample was measured by Coomassie brilliant blue protein assay kit (Nanjing Jiancheng Bioengineering Institute, Nanjing, Jiangsu, China). The obtained results were normalized against total protein concentration in each sample for inter-sample comparison.

### Determination of jejunal gene expression levels

Total RNA was extracted from the jejunal mucosa in line with the instructions of manufacturer using Trizol Reagent (Vazyme Biotech Co., Ltd, Nanjing, China). The RNA concentration and purity were determined using a NanoDrop ND-1000 UV spectrophotometer (NanoDrop Technologies, Wilmington, DE, USA). After that, 1 μg of total RNA was reverse-transcribed into complementary DNA though a process including 15 min at 37°C and 5 s at 85°C by PrimeScript RT reagent kit (Vazyme Biotech Co., Ltd, China) following its protocols. The complementary DNA samples were amplified with the ChamQ SYBR qPCR Master Mix Kit (Vazyme Biotech Co., Ltd, China) according to the manufacturer’s requirement. Real-time polymerase chain reaction (PCR) was carried out on a QuantStudio 5real-time PCR Design & Analysis system (Applied Biosystems, Carlsbad, CA, USA). The process of PCR was consisted of a pre-run at 95°C for 30 s, 40 cycles of denaturation at 95°C for 5 s, an annealing step at 60°C for 30 s and the melt-curve stage (95°C for 15 s, 60°C for 1 min and 95°C for 15 s). The standard curve of each gene was run in duplicate and three times for obtaining reliable amplification efficiency. The relative levels of mRNA expression were calculated using 2^−ΔΔCt^ method after normalization against the reference gene, β-actin [22]. The values of the CON group were used as a calibrator. All primers were synthesized by Sangon Biotech Co., Ltd. (Shanghai, China) and the sequences were showed in Table 2.

### Statistical analysis

All obtained data were processed by Excel 2010 first, and then analyzed by one-way ANOVA procedure using Statistical Analysis System (SAS Institute, 2000) [23] followed by a Duncan’s multiple range test. An individual broiler from each replicate was regarded as the experimental unit for measured indicators. The orthogonal polynomial contrasts were used to determine the linear and quadratic effects of increasing levels of YH. Data were presented as means and their pooled standard errors. The differences were considered as statistically significant when p<0.05.

## RESULTS

### Growth performance

Our previous study has showed that dietary YH addition linearly and quadratically increased (linear and quadratic, p<0.05) the body weight gain and gain-to-feed ratio during the starter (1 to 21 d), growth (22 to 42 d), and overall (1 to 42 d) periods of broilers [20].

### Serum DAO activity

According to Table 3, supplementing YH, irrespective of its level, decreased (linear and quadratic, p<0.05) serum DAO activity of broilers on 42 d of age in comparison with the CON group.

### Jejunal morphology

The results of jejunal morphology are shown in Table 4. Dietary YH supplementation linearly and quadratically increased (p<0.05) jejunal VH/CD on 21 d, VH and VH/CD on 42 d but decreased (p<0.05) jejunal CD on 21 d. Moreover, jejunal CD showed a linear reduction on 42 d (p<0.05). The highest VH and VH/CD values as well as lowest CD value both on 21 d and 42 d were observed in YH2 group.

### Jejunal inflammatory cytokines

In Table 5, compared with the CON group, the increased jejunal IL-10 concentration both on 21 d and 42 d was observed in YH2 and YH3 groups (linear and quadratic, p<0.05). Besides, jejunal TNF-α content was linearly decreased (p<0.05) on 21 d and quadratically reduced (p<0.05) on 42 d by YH supplementation.

### Jejunal barrier gene expression levels

Table 6 indicated that supplementing YH linearly and quadratically decreased (p<0.05) mucin 2 (*MUC2*) gene expression level whereas enhanced (p<0.05) zonula occludens-1 (*ZO-1*) gene expression level in the jejunum both on 21 d and 42 d compared with the CON group.

### Jejunal inflammatory gene expression levels

In Table 7, compared with the CON group, on 21 d of age, supplementing YH linearly and quadratically increased (p< 0.05) *IL-10* gene expression level whereas decreased (p<0.05) *IL-1β*, *TNF-α*, and myeloid differentiation factor 88 (*MyD88*) gene expression levels in the jejunum. On 42 d of age, jejunal IL-6 and nuclear factor kappa B (*NF-κB*) gene expression levels were linearly and quadratically decreased (p<0.05) whereas that of IL-10 showed an opposite result (p<0.05) by YH supplementation. Besides, jejunal *MyD88* gene expression level on 42 d was quadratically decreased (p<0.05) by YH inclusion.

## DISCUSSION

Recent research suggested that diets supplemented with brewer’s YH had beneficial effects on growth performance in finishing pigs [7]. Similarly, our previous study had found that YH supplementation could improve the body weight gain but decrease the gain to feed ratio during the starter, grower, and overall period of broilers [20]. The growth performance might be affected by many factors. Referring to our previous study, dietary YH supplementation enhanced intestinal digestive enzyme activities, antioxidant, and immune function of broilers [19,20]. Moreover, we could conclude that the positive effects of YH on growth performance might be also related to the improvement of intestinal morphology, barrier and anti-inflammatory functions of broilers based on this study.

The villus of small intestine is the main site for digestion and absorption in the body. Thus, the health of small intestine is vital to intestinal development, nutrient utilization, immune system, and the homeostasis of gut microbiota [24]. Two typical parameters, VH and CD, are always used to reflect the intestinal morphological development of animals. In addition, the greater value of VH/CD suggests excellent intestinal development and better ability to digest and absorb nutrients [25]. In our study, we firstly found that dietary YH supplementation at the level ranged from 100 to 150 mg/kg improved jejunal VH and VH/CD whereas decreased CD of broilers, and 100 mg/kg additional level presented better effects than 150 mg/kg. These findings suggested that YH addition efficiently improved intestinal morphology of broilers. Similar with our results, Fu et al [26] presented that supplementing YH increased jejunal VH and VH/CD as well as gut development-related genes expression to maintain intestinal integrity in weaned piglets. A previous study showed that dietary *Saccharomyces cerevisiae* hydrolysate increased the villus length and muscle thickness in the foregut and midgut as well as intestinal microvillus length and density in aquatic animals, thus increasing the intestinal absorptive surface area and improving nutrient capture [6], which might help to improve feed efficiency. Besides, other yeast-derived additives including live yeast and yeast culture also showed positive effects on intestinal morphology [13,27]. The beneficial effects of YH on intestinal morphology might in connection with its abundant nucleotides. It has been shown that nucleotides could promote intestinal development though facilitating the proliferation and maturation of intestinal cells [28]. Also, yeast cell wall components including MOS or β-glucan could dampen the pathogens (*Salmonella*, *Escherichia coli*, and *Listeria*) from adhering and colonizating in the intestine as well as interact with immune cells so as to neutralize intestinal villi atrophy due to inflammation [29,30]. Relevant experimental results reported that supplementing MOS and β-glucan enhanced small intestinal goblet cells count, increase VH and VH/CD but decreased CD in animals, thus improving intestinal morphology as well as benefiting intestinal health and growth performance ultimately [31,32]. Thus, the beneficial effects of supplementing YH might be related to the abundant bioactive agents to providing a healthy enteric environment for broilers.

The DAO expressed in intestinal mucosa upper chorial cells will release into blood circulation from the villus tips when intestinal barrier is impaired. Thus, serum DAO activity could be deemed as a blood marker to monitor the extent of intestinal damage [1]. Available evidence has showed that the damaged intestinal mucosa was always accompanied by an increased serum DAO activity [1,13]. In this study, dietary YH supplementation at the level ranged from 50 to 150 mg/kg decreased serum DAO activity in broilers, which suggested a decrease of intestinal permeability. Consistently, supplementing YH and other yeast-derived additives decreased serum endotoxin content and DAO activity in various animal studies [13,26,27]. The intestinal permeability is correlated with its intact mechanical barrier. Tight junction proteins act as a fence to prevent the translocation of macromolecular including harmful pathogenic bacteria and toxic substances by forming a paracellular permeability barrier. ZO-1, a crucial intestinal tight junction protein, which effectively defends against exogenous infections, thus maintaining the integrity of the intestinal barrier. Evidence has indicated that dietary YH supplementation protected intestinal mechanical barrier though up-regulating jejunal ZO-1 mRNA expression level in piglets [26]. Besides, Ducray et al [33] found that supplementing yeast fermentate prebiotic reversed the decreases of intestinal tight junction proteins expression including ZO-1, occludin (OCLN), and claudin (CLDN) in rats during heat stress though modulating the gut microbiota. Similarly, the enhanced jejunal *ZO-1* gene expression level by YH addition in the present study suggested the positive effects of YH on intestinal barrier function in broilers. Mucin 2 is the major component of the chemical barrier, which protects against the invasion of bacteria and pathogen as well as promotes intestinal restitution [34]. In the present study, jejunal *MUC2* gene expression was decreased by YH addition. Zhang et al [35] and Chen et al [36] showed that the MUC2 mRNA expression level was elevated in broilers challenged with lipopolysaccharide. The reason might be the existence of a kappa B site in the 5′-flanking region of the *MUC2* gene which activates *MUC2* gene to transcription level and enhances pro-inflammatory cytokine contents [37]. Various active substances contained in YH might be contribute to the improved intestinal barrier function. It has been reported that dietary prebiotics and probiotic could up-regulate intestinal tight junction proteins expression in animals [38]. Supplementing yeast nucleotide up-regulated ileal *ZO-1* and *CLDN* genes expression to increase the integrality of mechanical barrier in chickens [34]. Wang et al [13] and Han et al [29] demonstrated that dietary addition with yeast cell wall components, MOS and β-glucan, could up-regulate intestinal tight junction proteins expression, thus decreasing the intestinal permeability and improving the intestinal structural integrity in animals. Recent research indicated that the enhanced intestinal barrier function might be related to improved intestinal morphological structure [13], which coincided with our experimental results. These findings suggested that supplementing YH confers a beneficial effect on intestinal mucosal function and intestinal environment of broilers.

The control of intestinal inflammation is the key to healthy breeding. In the present study, dietary YH supplementation improved intestinal anti-inflammatory function of broilers, as evidenced by the increased IL-10 and reduced TNF-α concentration in the jejunum of broilers. Interleukin 10, secreted by activated macrophages, is a key cytokine to restrain the excessive production of pro-inflammatory cytokines and maintain immune balance of the body [13]. Conversely, TNF-α is a pro-inflammatory cytokine mainly produced by macrophages and monocytes, which level increases in many pathological conditions [5,6]. An article reported that lower serum immunoglobulins A and E levels and higher intestinal TNF-α level were observed in *IL-10* gene knockout mice compared with the mice without knockout [39]. Consistent with our results, dietary YH addition elevated serum IL-10 level but decreased IL-1β and TNF-α concentrations in pigs [16,26]. Waititu et al [4] indicated that yeast extract addition neutralized the increase of serum TNF-α and the decrease of IL-10 cytokines levels in weaned pigs challenged with *Escherichia coli* lipopolysaccharide, meanwhile, enhancing IL-10 but reducing *TNF-α* and *IL-1β* genes expression. From gene transcription levels, the jejunal *IL-6*, *1L-1β*, *TNF-α*, *NF-κB*, and *MyD88* genes expression were decreased while that of IL-10 was enhanced by YH supplementation in this study. IL-6, as a crucial mediator, could increase endothelial permeability by changing the ultrastructural of ZO-1 [40]. Additionally, IL-1β, IL-6 and TNF-α are essential cytokines to initiate inflammatory responses. Song et al [40] reported that NF-κB plays a key role in inducing intestinal inflammation via synthesizing and releasing pro-inflammatory cytokines including IL-6, 1L-1β, and TNF-α. Similarly, MyD88 exerts effects on toll-like receptor signal transduction pathways to induce various inflammatory responses and diseases. In coincidence with our results, dietary YH reduced intestinal TNF-α, 1L-1β, TLR4, and alkaline phosphatase genes expression to dampen the inflammatory response in different animals [6,26]. Evidence has been proposed that yeast culture addition down-regulated pro-inflammatory cytokines expression via restraining the pathway related to NF-κB in animals [3,27,41]. Also, Yuan et al [42] reported that supplementing a yeast-derived additive modulated the uterine inflammatory signals by decreasing uterine IL-6 mRNA expression as well as enhancing neutrophil myeloperoxidase and neutrophil elastase genes expression in transition dairy cows to reduce the incidence of subclinical endometritis. The active components in YH might be responsible for its anti-inflammatory function. Previous research indicated that the presence of *Saccharomyces cerevisiae* var. *boulardii* reduced intestinal inflammation and colonization by *Candida albicans* [43]. Dietary yeast nucleotides modulated gastrointestinal function and optimized intestinal microbiota to improve immunity function of intestinal mucosa [34]. The two main components in yeast cell wall, MOS and β-glucans, have also been shown to have beneficial anti-inflammatory effects. Dietary MOS supplementation enhanced serum IL-10 concentration but reduced IL-2, IL-4, and IFN-γ cytokines levels as well as intestinal *TLR2*, *TLR4*, *IL-8*, and *NF-κB* genes expression to suppress intestinal and systemic inflammation in sows [15]. An *in vitro* study indicated that yeast cell wall and β-glucans could activate CD22 receptors, which regulate the mitogen-activated protein kinase pathway and negatively regulate B cell activation to control the inflammatory response [44]. Likewise, it has been reported that β-glucans act as immunomodulators to weaken the development of inflammatory disease [45]. However, due to the intricacy nutrient composition in YH and the unclear mechanism by which YH improves the anti-inflammatory function of broiler chickens, further research on YH is still needed.

## CONCLUSION

It can be concluded that, the addition of 100 to 150 mg/kg YH generally improved intestinal morphology, barrier and anti-inflammatory functions whereas decreased intestinal permeability of broilers.

## Figures and Tables

**Figure 1:**
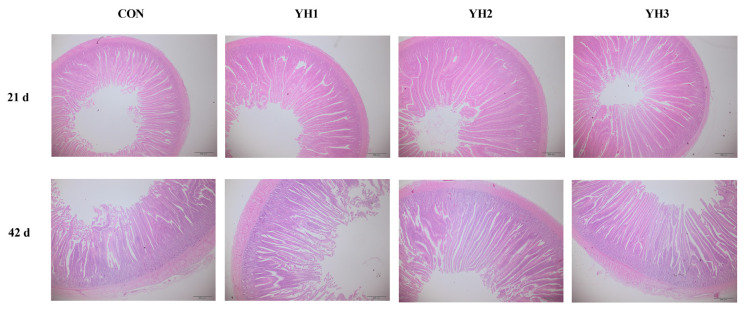
Haematoxylin and eosin (H&E) staining of jejunum on 21 and 42 days of age of broilers. Scale bar, 500 μm. CON, basal diet; YH1, YH2, and YH3 group, basal diet adding 50, 100, and 150 mg/kg YH, respectively.

**Table 1:** Composition and nutrient content of the basal diet (as fed basis)

Item	1 to 21 d	22 to 42 d
Ingredient (%)		
Corn	55.60	55.20
Soybean meal (44%, crude protein)	29.00	24.00
Cottonseed meal (44%, crude protein)	2.50	3.00
Wheat flour	4.00	4.00
hydrolyzed feather meal	1.50	1.50
Dicalcium phosphate	0.90	0.80
Limestone	1.50	1.50
Amargosite	1.00	1.00
Soybean oil	2.00	7.00
Premix^1)^	2.00	2.00
Calculated nutrient levels		
Metabolisable energy (MJ/kg)	12.46	13.38
Crude protein (%)	21.50	19.51
Calcium (%)	0.96	0.84
Total phosphorus (%)	0.66	0.55
Lysine (%)	1.45	1.40
Methionine (%)	0.54	0.50
Threonine (%)	0.91	0.80

1) The premix provided per kilogram of diet: vitamin A, 12,000 IU; vitaminD_3_, 2,500 IU; vitamin E, 20 IU; menadione sodium bisulfate, 1.3 mg; thiamin, 2.2 mg; riboflavin, 8.0 mg; nicotinamide, 40 mg; calcium pantothenate, 10 mg; choline chloride, 400 mg; pyridoxine HCl, 4 mg; biotin, 0.04 mg; folic acid, 1 mg; vitamin B_12_ (cobalamin), 0.013 mg; Fe (from ferrous sulfate), 80 mg; Cu (from copper sulfate), 8 mg; Mn (from manganese sulfate), 110 mg; Zn (from zinc sulfate), 60 mg; I (from calcium iodate), 1.1 mg; Se (from sodium selenite), 0.3 mg.

**Table 2:** Primer sequences used for the real-time polymerase chain reaction analysis

Gene	Genbank number	Primer sequences (5′-3′)	Product size (bp)
*β-actin*	NM_205518.1	Forward: TTGGTTTGTCAAGCAAGCGG	100
		Reverse: CCCCCACATACTGGCACTTT	
*IL-6*	NM_204628.1	Forward: AGGGCCGTTCGCTATTTGAA	72
		Reverse: CAGAGGATTGTGCCCGAACT	
*IL-10*	NM_001004414.2	Forward: GGAGCTGAGGGTGAAGTTTGA	129
		Reverse: GACACAGACTGGCAGCCAAA	
*IL-1β*	NM_204524.1	Forward: GTACCGAGTACAACCCCTGC	112
		Reverse: AGCAACGGGACGGTAATGAA	
*TNF-α*	JN_942589	Forward: AGACCAGATGGGAAGGGAATGAA	219
		Reverse: GAAGAGGCCACCACACGACAG	
*IFN-γ*	NM_205149.1	Forward: CACTGACAAGTCAAAGCCGC	87
		Reverse: ACCTTCTTCACGCCATCAGG	
*TLR4*	NM_001030693.1	Forward: AGGCACCTGAGCTTTTCCTC	96
		Reverse: TACCAACGTGAGGTTGAGCC	
*NF-κB*	NM_205129.1	Forward: GTGTGAAGAAACGGGAACTG	203
		Reverse: GGCACGGTTGTCATAGATGG	
*MyD88*	NM_001030962.1	Forward: ATCCGGACACTAGAGGGAGG	115
		Reverse: GGCAGAGCTCAGTGTCCATT	
*OCLN*	NM_205128.1	Forward: CCGTAACCCCGAGTTGGAT	214
		Reverse: ATTGAGGCGGTCGTTGATG	
*ZO-1*	XM_413773.4	Forward: TGTAGCCACAGCAAGAGGTG	159
		Reverse: CTGGAATGGCTCCTTGTGGT	
*MUC2*	XM_001234581.3	Forward: AGGAATGGGCTGCAAGAGAC	77
		Reverse: GTGACATCAGGGCACACAGA	
*CLDN2*	NM_001277622.1	Forward: CCTGCTCACCCTCATTGGAG	145
		Reverse: GCTGAACTCACTCTTGGGCT	

*IL-6*, interleukin-6; *IL-10*, interleukin-10; *IL-1β*, interleukin-1β; *TNF-α*, tumor necrosis factor-alpha; *IFN-γ*, interferon γ; *TLR4*, toll-like receptor 4; *NF-κB*, nuclear factor kappa B; *MyD88*, myeloid differentiation factor 88; *OCLN*, occludin; *ZO-1*, zonula occludens-1; *MUC2*, mucin 2; *CLDN2*, claudin 2.

**Table 3:** Effects of yeast hydrolysate on serum diamine oxidase activity of broilers

Item	Dietary treatment^1)^				SEM^2)^	p-value	
	
	CON	YH1	YH2	YH3		Linear	Quadratic
21 d							
DAO (U/L)	39.984	37.664	35.212	39.228	1.103	0.697	0.572
42 d							
DAO (U/L)	48.598^b^	40.707^a^	38.912^a^	37.234^a^	1.018	0.011	0.002

DAO, diamine oxidase.

1) CON, basal diet; YH1, YH2, and YH3 group, basal diet adding 50, 100, and 150 mg/kg YH, respectively.

2) Standard error of the means (n = 8).

a,b Means within the same row with no common superscript differ significantly (p<0.05).

**Table 4:** Effects of yeast hydrolysate on jejunal morphology of broilers

Item	Dietary treatment^1)^				SEM^2)^	p-value	
	
	CON	YH1	YH2	YH3		Linear	Quadratic
21 d							
VH (μm)	755.651	756.832	813.188	794.199	18.874	0.121	0.281
CD (μm)	98.642^a^	92.652^ab^	84.651^b^	86.712^b^	2.265	0.002	0.004
VH/CD	7.581^c^	7.929^bc^	9.362^a^	8.981a^b^	0.764	0.003	0.009
42 d							
VH (μm)	1,467.524^c^	1,512.538^bc^	1,635.886^a^	1,526.320^b^	27.987	0.017	<0.001
CD (μm)	198.641^ab^	200.087^bc^	188.463^c^	190.131^c^	3.926	0.022	0.074
VH/CD	7.409^c^	7.574^c^	8.692^a^	8.034^b^	0.665	0.001	<0.001

VH, villus height; CD, crypt depth; VH/CD, the ratio of villus height to crypt depth.

1) CON, basal diet; YH1, YH2, and YH3 group, basal diet adding 50, 100, and 150 mg/kg YH, respectively.

2) Standard error of the means (n = 8).

a–c Means within the same row with no common superscript differ significantly (p<0.05).

**Table 5:** Effects of yeast hydrolysate on jejunal inflammatory cytokines of broilers

Item	Dietary treatment^1)^				SEM^2)^	p-value	
	
	CON	YH1	YH2	YH3		Linear	Quadratic
21 d							
IL-10 (ng/g protein)	5.590^b^	6.507^ab^	7.632^a^	7.589^a^	0.223	0.025	0.045
TNF-α (ng/g protein)	9.614^a^	8.340^ab^	8.084^b^	8.441^ab^	0.362	0.039	0.051
42 d							
IL-10 (ng/g protein)	5.988^b^	7.014^ab^	7.550^a^	7.314^a^	0.171	0.019	0.017
TNF-α (ng/g protein)	9.445^a^	8.799^ab^	7.733^b^	9.122^a^	0.163	0.291	0.039

IL-10, interleukin-10; TNF-α, tumor necrosis factor-alpha.

1) CON, basal diet; YH1, YH2, and YH3 group, basal diet adding 50, 100, and 150 mg/kg YH, respectively.

2) Standard error of the means (n = 8).

a,b Means within the same row with no common superscript differ significantly (p<0.05).

**Table 6:** Effects of yeast hydrolysate on jejunal barrier genes expression of broilers

Item	Dietary treatment^1)^				SEM^2)^	p-value	
	
	CON	YH1	YH2	YH3		Linear	Quadratic
21 d							
MUC2	1.000^a^	0.508^b^	0.501^b^	0.573^b^	0.045	0.022	0.003
OCLD	1.000	0.968	1.009	0.986	0.044	0.996	0.998
ZO-1	1.000^a^	1.513^b^	1.663^b^	1.518^b^	0.052	0.006	0.011
CLDN2	1.000	1.153	1.222	1.219	0.043	0.160	0.305
42 d							
MUC2	1.000^a^	0.698^b^	0.585^b^	0.581^b^	0.034	0.003	0.003
OCLD	1.000	0.983	0.878	0.980	0.036	0.867	0.979
ZO-1	1.000^a^	1.136^ab^	1.321^b^	1.360^b^	0.061	0.043	0.026
CLDN2	1.000	1.017	1.043	1.013	0.052	0.504	0.336

MUC2, mucin 2; OCLN, occludin; ZO-1, zonula occludens-1; CLDN2, claudin 2.

1) CON, basal diet; YH1, YH2, and YH3 group, basal diet adding 50, 100, and 150 mg/kg YH, respectively.

2) Standard error of the means (n = 8).

a,b Means within the same row with no common superscript differ significantly (p<0.05).

**Table 7:** Effects of yeast hydrolysate on jejunal inflammation-related genes expression of broilers

Item	Dietary treatment^1)^				SEM^2)^	p-value	
	
	CON	YH1	YH2	YH3		Linear	Quadratic
21 d							
IL-6	1.000	0.762	0.735	0.887	0.061	0.497	0.204
IL-10	1.000^b^	1.584^a^	2.137^c^	1.885^a^	0.115	0.017	0.019
IL-1β	1.000^a^	0.834^ab^	0.780^ab^	0.603^b^	0.044	0.001	0.006
TNF-α	1.000^a^	0.679^b^	0.527^c^	0.648^b^	0.052	0.014	0.005
IFN-γ	1.000	0.968	0.988	0.903	0.055	0.611	0.852
TLR4	1.000	1.016	0.920	0.977	0.058	0.715	0.917
NF-κB	1.000	1.100	1.024	0.956	0.040	0.370	0.272
MyD88	1.000^a^	0.736^ab^	0.681^b^	0.640^b^	0.045	0.005	0.016
42 d							
IL-6	1.000^a^	0.700^b^	0.534^b^	0.661^b^	0.082	0.018	0.008
IL-10	1.000^a^	1.195^ab^	1.453^b^	1.637^b^	0.189	0.032	0.028
IL-1β	1.000	0.844	0.753	0.734	0.064	0.082	0.187
TNF-α	1.000	0.969	0.879	0.806	0.049	0.131	0.319
IFN-γ	1.000	0.954	0.880	1.011	0.033	0.430	0.296
TLR4	1.000	0.928	0.823	0.912	0.049	0.448	0.572
NF-κB	1.000^a^	0.912^ab^	0.904^b^	0.826^b^	0.034	0.033	0.001
MyD88	1.000^a^	0.931^ab^	0.891^ab^	0.799^b^	0.047	0.069	0.018

IL-6, interleukin-6; IL-10, interleukin-10; IL-1β, interleukin-1β; TNF-α, tumor necrosis factor-alpha; IFN-γ, interferon γ; TLR4, toll-like receptor 4; NF-κB, nuclear factor kappa B; MyD88, myeloid differentiation factor 88.

1) CON, basal diet; YH1, YH2, and YH3 group, basal diet adding 50, 100, and 150 mg/kg YH, respectively.

2) Standard error of the means (n = 8).

a–c Means within the same row with no common superscript differ significantly (p<0.05).
